# Supplementary material for: Parietal alpha tACS shows inconsistent effects on visuospatial attention
Source: PLoS One. 2021 Aug 5;16(8):e0255424. doi: 10.1371/journal.pone.0255424 (PMC8341497; doi:10.1371/journal.pone.0255424)
Supplement: Attachment — Submitted filename: Response to Reviewers.docx [file pone.0255424.s001.docx]

**RESPONSE TO DECISION LETTER**

Dear Editor,

We are grateful to the editors and reviewers for taking the time to read and provide constructive suggestions for improvement of our manuscript. We have revised our manuscript accordingly, taking into consideration all comments. Below we address all the comments point-by-point and explain how we have integrated them. Changes to the original text are highlighted in the manuscript using bold and underline.

Yours Sincerely,

On behalf of the co-authors

Andra Coldea

**Reviewer’s comments to Authors**

**Reviewer 1**

Coldea and colleagues show the inconsistency of parietal alpha tACS to modulate visuospatial attention. They replicated a previous paradigm from Schuhmann and colleagues (2019), where after a cue, participants had to indicate whether a gabor patch was oriented clockwise or counterclockwise.
The authors were not able to replicate previous findings, however they did not mirror completely Schuhmann and colleagues (2019) stimulation parameters, in fact they stimulated for 20 instead of 40 minutes and using higher intensity.

General Comment

I like this article. It is easy to follow and I enjoyed reading it. I truly think that null results must be published. However, I have some comments (that need to be addressed in my opinion) and that may help the authors to improve the current version of the manuscript. I did also some suggestion that the authors may ignore if they find them not interesting.

Author response: Thank you for your appreciation.

**Introduction**
Comment
Lines 58-65. Sorry, I do not understand this sentence. You say occipito-parietal tACS at alpha frequency bias perception in a spatially specific manner. Why is it an indirect support that tACS causally interact with attention-related brain oscillation? Please clarify or expand. (as it is, I do not agree with this sentence, perception and attention are two distinct processes, you can modulate one, without modulating the other process. you do not have to agree with me, just clarify and expand this sentence or change it).

Author response: Thank you for your observation. This sentence has been changed, and the text on page 4 now reads: “In the context of visuospatial attention, if occipito-parietal tACS at alpha-frequency were to bias behavioural performance in a spatially specific manner, this would be in (indirect) support of tACS causally interacting with underlying, perceptually relevant brain oscillations.”

Moreover, I would strongly suggest to expand the sentences “in addition many EEG/MEG- studies have established… alpha power and perception (most of the paper you cited are about awareness, confidence [instead of perceptual accuracy], response criterion etc. are you sure that you can say that there is a link between alpha power and perception in general?).

Author response: Thank you for your suggestion. This sentence has been changed, and the text on page 4 now reads: “In addition, many EEG/MEG-studies have established a link between posterior alpha-power and specific behavioural outcomes in perceptual tasks, such as perceptual accuracy (20,26–29) or subjective awareness of visual stimuli (30–35).”

I would be more specific, I think that is established the link between posterior alpha power and TEMPORAL INTEGRATION of visual stimuli. There are a lot of paper about this topic, not only EEG/MEG studies, but also tACS studies and even studies that use sensory entrainment paradigm.

**EEG/MEG studies**

- Samaha, J., & Postle, B. R. (2015). The speed of alpha-band oscillations predicts the temporal resolution of visual perception. Current Biology, 25(22), 2985-2990.

- VanRullen, R. (2016). Perceptual cycles. Trends in cognitive sciences, 20(10), 723-735.
- Ronconi, L., Oosterhof, N. N., Bonmassar, C., & Melcher, D. (2017). Multiple oscillatory rhythms determine the temporal organization of perception. Proceedings of the National Academy of Sciences, 114(51), 13435-13440.

tACS (Ghiani et al., ( 2021) is an interesting review about tACS and temporal or spatial integration of visual event)
- Battaglini, L., Mena, F., Ghiani, A., Casco, C., Melcher, D., & Ronconi, L. (2020). The effect of alpha tACS on the temporal resolution of visual perception. Frontiers in psychology, 11, 1765.
- Ghiani, A., Maniglia, M., Battaglini, L., Melcher, D., & Ronconi, L. (2021). Binding mechanisms in visual perception and their link with neural oscillations: a review of evidence from tACS. Frontiers in Psychology, 12, 779.

- Cecere, R., Rees, G., and Romei, V. (2015). Individual differences in alpha frequency drive crossmodal illusory perception. Curr. Biol. 25, 231–235.

**tACS with focus on the phase**

- Ronconi, L., Melcher, D., Junghöfer, M.,Wolters, C. H., and Busch, N. A. (2020). Testing the effect of tACS over parietal cortex in modulating endogenous alpha rhythm and temporal integration windows in visual perception. Europ. J. Neurosci. doi: 10.1111/ejn.15017

**Sensory entrainment**

- Ronconi, L., & Melcher, D. (2017). The role of oscillatory phase in determining the temporal organization of perception: evidence from sensory entrainment. Journal of Neuroscience, 37(44), 10636-10644.
- Ronconi, L., Busch, N. A., & Melcher, D. (2018). Alpha-band sensory entrainment alters the duration of temporal windows in visual perception. Scientific reports, 8(1), 1-10.

Author response: Thank you for your comment and these important recommendations from the literature. The studies cited in our earlier version and present introduction are primarily related to posterior alpha power and spatial bias in perception, as this was the focus of our study and the design replication. The studies that are suggested here are more about temporal aspects of perception (temporal resolution, binding) and also include alpha frequency and phase (not only power which was our focus) as a possible mechanistic explanation of these perceptual processes.

In order not to distract from our primary focus in the introduction but to acknowledge the above important literature, we have integrated many of the papers above and also the points related to temporal integration in the discussion on page 28, which now reads: “Here, we demonstrate variable effects of transcranial electrical stimulation techniques when they are targeting alpha oscillations for the purpose of modulating visuospatial attention in healthy participants. However, tACS has been successfully used for modulating alpha and beta oscillations in relation to other visual processes, such as temporal (57–61) and spatial binding (62). Our study and the negative results should hence not be taken to generalize to other relationships between brain oscillations and perceptual processes and their tests through non-invasive brain stimulation techniques.”

**Materials and method**

Suggestion:
Please report current density if possible, (it is more important than mA, you can have the same current density with different electrodes size and different intensities). It would be interesting to see a pictures about voltage distribution if possible.

Author response: Thank you for bringing this important point to our attention. We have now included the information regarding the current density. This has now been changed in the text on page 11 to read: “High-density tACS was delivered through a battery-driven, constant current stimulator (NeuroConn GmbH, Ilmenau, Germany) using a rubber ring tACS electrode with a small central, circular electrode (2.1 cm diameter, 3.5 cm2; thickness: 2 mm) and a large outer ring (9 cm inner and 11 cm outer diameter, 31.5 cm2; thickness: 2 mm) (as in 36). […] Electrode impedance was kept below 10 kΩ. Stimulation frequency was 10Hz (as in 36) but the intensity was set slightly higher to 1.5 mA (peak-to-peak), yielding an average current density of 0.4 mA/cm2 at the central electrode, and 0.05 mA/cm2 at the surround electrode. For a picture with the simulated voltage distribution, we refer to (36), their Fig 1A.”

**Results**
Why don’t you run an ANCOVA (IAF would be the covariate) when you want to see whether the participants’ performance depends on participants’ IAF instead of grouping participants in an arbitrary way (according to how much their IAF differed from 10 Hz)? See the analysis conducted by Battaglini et al,. (2020), frontiers in Psychology. Think about it.

- Battaglini, L., Mena, F., Ghiani, A., Casco, C., Melcher, D., & Ronconi, L. (2020). The effect of alpha tACS on the temporal resolution of visual perception. Frontiers in psychology, 11, 1765.

Author response: Thank you for your excellent suggestion. We agree that an ANCOVA would add more value (compared to our previous analysis in which we grouped participants in two groups), and we have now updated the existing analysis using your suggestion, in the corresponding sections of the method (page 14/15) and results. To remain congruent throughout our manuscript, we included the ANCOVA not only for the analysis of IAF (as suggested), but also for the analyses of whether individual spatial bias or individual alpha power may have influenced the tACS outcome (see sections: Dependency of tACS-effects (RT) on trait factors: Individual spatial bias and alpha-frequency/power, page 20 onwards).

We believe that this also simplified the reading as, instead of using as our dependent variable the stimulation effect as we did previously (calculated as 10 Hz tACS (RT_Left_ – RT_Right_) – Sham (RT_Left_ – RT_Right_)), we can now mirror the main analysis using tACS condition and cue validity as factors, where our dependent measure was the hemifield bias, and add the covariates. We thus ran four ANCOVAs, using individual bias, IAF, deviation of IAF from 10Hz, and alpha power as covariates. For none of these ANCOVAs did we observe any statistically significant evidence for the covariates influencing tACS outcome (no interaction of the covariate with tACS or with tACS and cue validity). This differs in one instance from our previous group analysis, where we found a significant interaction between group (IAF below vs above 10Hz stimulation frequency) and tACS outcome. However, because this interaction was a trivial effect (as most likely driven by regression to the mean, see former version) and because we agree that the analysis including a covariate is more appropriate, we decided to stick to the ANCOVA result. As a result, we have now also taken out former Fig4 (that illustrated the effect of individual bias on outcome) and changed the discussion accordingly.

**Discussion**
Suggestion:
My feeling is that this paper tells us only a part of the tACS story. You focused only in papers that report visuospatial tACS effect and that is ok since it is the main topic. However, in the discussion I would report that parietal cortex was often associated with segmentation process and local processing and that eventually tACS may modulate attentional process only when the task is linked with local processing. (in other words the effect of tACS on perception or attention is task specific). Please see:

- Zaretskaya, N., & Bartels, A. (2015). Gestalt perception is associated with reduced parietal beta oscillations. Neuroimage, 112, 61-69.

- Battaglini, L., Ghiani, A., Casco, C., & Ronconi, L. (2020). Parietal tACS at beta frequency improves vision in a crowding regime. Neuroimage, 208, 116451.

- Romei, V., Driver, J., Schyns, P. G., & Thut, G. (2011). Rhythmic TMS over parietal cortex links distinct brain frequencies to global versus local visual processing. Current biology, 21(4), 334-337.
Moreover, there is also an interesting study that indicate that parietal cortex is more sensitive to beta activity rather than alpha

- Samaha, J., Gosseries, O., and Postle, B. R. (2017). Distinct oscillatory frequencies underlie excitability of human occipital and parietal cortex. J. Neurosci. 37, 2824–2833. doi: 10.1523/JNEUROSCI.3413-16.2017

Author response: We agree and have therefore added the following sentence to the newly added discussion section on alpha and beta oscillations and temporal and spatial binding (page 28): “Our study and negative results should however not be taken to generalize to other relationships between brain oscillations and perceptual processes and their tests through non-invasive brain stimulation techniques.”

For the full newly added discussion section, please see pages 27 onwards.

Comment
I found interesting the difference in stimulation intensity and duration between yours and Schuhmann and colleagues’ study. Please consider the phenomenon of stochastic resonance, it works only when the appropriate level of noise is introduced in the system. When you insert too much noise the phenomenon disappears. Analogously, could be that you have inserted in the system too much noise stimulating at 1.5mA? If you calculate the current density (that is more important than mA) you can find out whether the current that you “inserted in the brain” is equal or greater than previous papers
(current density = mA/cm^2).

Author response: Following the reviewer’s suggestion, the current density calculation has now been integrated in the paper (please see one of the comments above). We agree that it is possible that the inconsistent results between our and previous studies could be explained out-width an entrainment framework, such as by the phenomenon of stochastic resonance. However, one of the studies we cite on alpha tACS and spatial bias, namely Kemmerer et al. (2020), reported an online effect of tACS on endogenous attention using the exact same electrodes and setup as us, and at a stimulation intensity of 1.5 mA. Considering these results, interpreting our negative findings in line with the phenomenon of stochastic resonance might be a bit too speculative (also because it would call into question the entrainment explanation favoured by all the cited tACS studies on visuospatial attention, in favour of different amounts of noise having been injected). In general, we are not very comfortable adding too many speculations based on our negative results. Instead, we tried to keep our paper more descriptive to avoid (over)interpretations of our null result.

Comment
It would be interesting to see how long stimulated previous papers, perhaps tACS need time to produce effect. To the best of my knowledge the papers below (topic: vision and attention) got an effect stimulating for about 40 min.

- Kasten, F. H., Wendeln, T., Stecher, H. I., & Herrmann, C. S. (2020). Hemisphere-specific, differential effects of lateralized, occipital–parietal α-versus γ-tACS on endogenous but not exogenous visual-spatial attention. Scientific reports, 10(1), 1-11.

- Battaglini, L., Mena, F., Ghiani, A., Casco, C., Melcher, D., & Ronconi, L. (2020). The effect of alpha tACS on the temporal resolution of visual perception. Frontiers in psychology, 11, 1765.
- Schuhmann, T., Kemmerer, S. K., Duecker, F., De Graaf, T. A., Ten Oever, S., De Weerd, P., & Sack, A. T. (2019). Left parietal tACS at alpha frequency induces a shift of visuospatial attention. PLoS One, 14(11), e0217729.

Author response: Thank you for your comment. Indeed, we have included in our manuscript two of the three papers the reviewer recommends (Kasten et al., 2020 & Schuhmann et al., 2019), as these papers directly address endogenous visuospatial attention. We also cited all other published papers addressing the same topic and listed them in the table on pages 6-7 of our manuscript. In summary, an effect of alpha tACS on endogenous attention in the visual domain has been found with tACS durations ranging from 8 minutes per block (Kasten et al., 2020) to 40 minutes per block (Schuhmann et al.,2019; Kemmerer et al.,2020). In the auditory domain, tACS effects on endogenous attention have been found with durations similar to the ones implemented in our study, i.e. of 20-25 minutes (Woestmann et al., 2018; Deng et al., 2019). Hence, based on these studies, stimulation duration seems to be a poor predictor of outcomes of alpha tACS on spatial perceptual bias.

**Reviewer 2**

The present study of Coldea et al., entitled “Parietal alpha tACS shows inconsistent effects on visuospatial attention”, investigated the behavioural effects of alpha-tACS on spatial attention. Their primary goal is to replicate the reported modulation of spatial attention with alpha-tACS applied to the parietal cortex. They recruited 40 healthy participants who underwent left parietal tACS during task performance in two separate sessions (10 Hz tACS or sham). However, their results indicated that left parietal tACS did not shift the bias to the left, as compared to sham, irrespective of cueing condition. It was an interesting article that will further our understanding of how tACS affects neuronal networks. The results themselves deserve publication because the consistency of brain stimulation after-effects is a matter of recent debate. However, there are some points that I want clarifications and suggestions that the authors may consider to improve the quality of the manuscript.

Author response: Thank you very much for your careful and constructive review.

**Methods**
1. On page 10, kindly (shortly) described how trials are considered “valid”, “invalid”, and “neutral”.

Author response: Thank you for your comment. The addition has been made on page 10, which now reads: “One experimental session consisted of 336 trials containing 192 valid trials (i.e. target was presented in the cued hemifield), 48 invalid trials (i.e. target was presented opposite the cued hemifield), and 96 neutral trials (i.e. target was preceded by a neutral cue).”

2. Indicate the stimulation duration in the “tACS section”.

Author response: Thank you for your comment. The addition has been made in the “tACS section”on page 11, which now reads: “In the active condition, phase offset was set to 0 at the start and 100 cycles were used for ramping up, with the stimulator being switched off after completion of the experimental task. The stimulation duration was approximately 20 minutes.”

3. “Participants were instructed to respond as fast and as accurately as possible, using the index and middle finger of their right hand”...did they use button presses? How is the reaction time (RT) defined?

Author response: Thank you for your comment. The addition has been made in the “Procedure and task” section on page 10, which now reads: “Participants had to discriminate whether the Gabor patch was oriented clockwise or counterclockwise and were instructed to respond as fast and as accurately as possible once the target appeared on the screen, by pressing the left and right arrow keys on the keyboard, using the index and middle finger of their right hand, respectively.”

**Data analysis**

1. Is the statistical analysis of the data entirely identical to Schuhmann et al., 2019? RTs are not normally distributed, did the authors considered using non-parametric tests or log-transformed them before performing their parametric tests (ANOVA)?

Author response: Thank you for your suggestion. Identical to Schuhmann et al. (36), due to the skewness of the reaction times, we used median RT scores per condition. However, we appreciate this might not be sufficient, so following your suggestion, the RTs were log-transformed and the analyses redone using the log-transformed data. This has rendered qualitatively identical results, not changing the conclusions (please see below for more details). We have added a clarification in the Data analyses section of Materials and Methods on page 13, which now reads: “For each participant, accuracy and median RTs were computed for each tACS condition (i.e. 10Hz tACS vs sham), type of cue (i.e. invalid, neutral, valid) and target location (i.e. left hemifield vs right hemifield), in analogy to (36). Because the RT distributions are usually skewed (43), we also conducted the analyses using the log-transformed data. The results of the analyses remained qualitatively unchanged, not affecting the conclusions, hence these analyses are not reported in the paper.”

Because we want to remain as close as possible to Schuhmann et al. (2019) for purposes of comparability, we stick in the paper to the reporting of the median RT analysis, but report the outcome of the log-transformation in some detail below for your convenience. When redoing the analyses using log-transformed data, we first checked whether the experimental manipulation of spatial attention was effective. Similar to the original analyses, we found a main effect of validity (F(2,78) = 40.6, p <.001, $\eta_{G}^{2}$=.03) and of hemifield (F(1,39) = 12.6, p = .001, $\eta_{G}^{2}$=.01) in the sham condition. Our main analyses, examining whether left parietal tACS induced a bias away from the right to the left hemifield when 10Hz tACS was applied, generated the following results when ran with log-transformed data:

Hence, the (in)significance of the results remains unchanged in comparison to the analysis of the median RTs.

**Results**
1. The authors reported a significant main effect of cue validity (Figure 2A). However, by looking in Figure 2A, we can clearly see that the differences between the valid trials (450.7±70.5ms), neutral trials (464±76ms) and invalid trials (485.7±85.8ms) are not robust. The overlapping SDs are also indicative. Are the post hoc comparisons corrected? For example Bonferroni? Kindly check your data for outliers.

Author response: Thank you for highlighting this point. The post hoc comparisons were indeed Bonferroni corrected, which has now been added to the text. The plots in former Figure 2A did indeed show a big overlap in SDs, which can be misleading though, as they include between-subject variability, whereas the repeated-measure ANOVA (rm-ANOVA) does parcel out this between-subject variability. Hence, there is the possibility of a discrepancy between variability as illustrated in error bars and variability across within-subject factors as analysed by an rm-ANOVA. For the purposes of visualisation of the data only, we have adjusted the standard error bars according to the alternative method suggested by Cousineau (2005), which takes into account the repeated-measures design used in the study. The figure has now been adjusted to reflect these changes. For clarity, we have also superimposed the individual data points on the plot. We hope this clarifies the source of the big overlap in error bars in the former Figure (which was between-subjects), while the data show high consistency within-subjects (translating into highly significant effects on the within-subject factor cue validity).

Cousineau, D. (2005). Confidence intervals in within-subject designs: A simpler solution to Loftus and Masson’s method. *Tutorials in Quantitative Methods for Psychology,* 1(1), p. 42-54.

2. I have the same opinion for Fig 2B. I would not assume significant differences between the hemifields. Why ANOVA? You are only comparing two factors, right and left hemifield.

Author response: The same considerations as above also apply for figure 2B, which has now been adapted following the abovementioned procedures. The significant difference between the hemifields is now reported using a paired t-test, and remain significant due to the consistency within subjects. The text on page 16 now reads as follows: “We then tested whether there was a difference between the RTs in the left as compared to the right hemifield in the sham condition (RT left > right visual field), as reported by Schuhmann et al. (2019) employing the same paradigm. A t-test on median RTs (data averaged across cue validity, Fig 2B) indeed revealed a significant difference between hemifields (t(39)=3.13, p =.003, r2=.24)”.

3. The analysis for the State-dependency of tACS-effects (on RT): Pre-existing spatial bias seems erroneous. The authors considered the change in spatial bias from sham to tDCS. But this scenario only validly applied for those participants that were stimulated with sham on the first session and tACS on the second session. I meant the “order effect” because the session was counterbalanced (sham-tACS vs tACS-sham). In my opinion, the two-day intersession interval is short, so we cannot rule out the cross-over effect. Therefore we cannot correctly consider the spatial bias from subjects with a tACS-sham session schedule as pre-existing. This comment also applies to the rest of the analysis in this section (IAF, amplitude).

Author response: Thank you for your comment. This is a valid point. Nonetheless, we feel it may be of interest for researchers in the field to know whether any of the behavioural effects may depend on a marker of individual trait bias. We are now approaching this more carefully through the following changes. We dropped the term ‘pre-existing bias’ and replaced it with ‘individual (trait) bias’ but acknowledge that our measure of individual bias is an estimate (from sham data), post-hoc and therefore suboptimal. The relevant section in the text on page 22 now reads: “We note though that our exploratory analyses of the impact of covariates was post-hoc, and our design not optimal for inferring individual trait factors, as inferred during sham (counterbalanced with tACS), when these should have ideally been inferred before any experimental manipulation”.

4. Of the 40 participants whose data were included in the analysis, 25 could distinguish between 10 Hz tACS and sham sessions correctly. This indicates that blinding was compromised in more than half of the participants. Is this comparable to the previous studies? I want advice analysing the date of the group which remained blinded.

Author response: Thank you for your comment. It is true that the blinding does not seem to have been successful in over half of our sample. However, 25/40 is very close to chance performance (according to chi-square goodness of fit this is not significantly different from chance 20/40, p=0.12), suggesting that across the group participants may have had to guess which session was which. In other words, as participants had 50% chance to correctly guess, concluding that half of our sample was not blind would seem an overestimate. But we acknowledge that blinding is a problem in tES studies as has been often pointed out (e.g. Horvath et al., 2014). In recent double-blind tDCS experiments, for instance, participants were able to distinguish between sham and active tDCS at intensities of 1 mA (Greinacher et al., 2019; Turi et al., 2019) and 2 mA (O’Connell et al., 2012). Moreover, in Schumann et al., a considerable amount of participants (around 40%) correctly identified the active and sham sessions. Therefore, we consider that the fact that many participants were able to correctly distinguish between the two sessions was not due to an experimental issue of our particular study, but rather reflects a concern endemic to all NIBS techniques. Accordingly, we believe the approach of analysing the whole sample (as practiced in the whole field) to be appropriate, and hope the reviewer agrees.

Greinacher, R., Buhôt, L., Möller, L., & Learmonth, G. (2019). The time course of ineffective sham-blinding during low-intensity (1 mA) transcranial direct current stimulation. European Journal of Neuroscience, 50(8), 3380–3388. https://doi.org/10.1111/ejn.14497

Horvath, J. C., Carter, O., & Forte, J. D. (2014). Transcranial direct current stimulation: Five important issues we aren’t discussing (but probably should be). Frontiers in Systems Neuroscience, 8. https://doi.org/10.3389/fnsys.2014.00002

O’Connell, N. E., Cossar, J., Marston, L., Wand, B. M., Bunce, D., Moseley, G. L., & Souza, L. H. D. (2012). Rethinking Clinical Trials of Transcranial Direct Current Stimulation: Participant and Assessor Blinding Is Inadequate at Intensities of 2mA. PLOS ONE, 7(10), e47514. https://doi.org/10.1371/journal.pone.0047514

Turi, Z., Csifcsák, G., Boayue, N. M., Aslaksen, P., Antal, A., Paulus, W., Groot, J., Hawkins, G. E., Forstmann, B., Opitz, A., Thielscher, A., & Mittner, M. (2019). Blinding is compromised for transcranial direct current stimulation at 1 mA for 20 min in young healthy adults. European Journal of Neuroscience, 50(8), 3261–3268. https://doi.org/10.1111/ejn.14403
